# Genomic stability of pulmonary artery endothelial colony-forming cells in culture

**DOI:** 10.1177/2045893217700901

**Published:** 2017-05-12

**Authors:** Kylie M. Drake, Chiara Federici, Heng T. Duong, Suzy A. Comhair, Serpil C. Erzurum, Kewal Asosingh, Micheala A. Aldred

**Affiliations:** 1 Genomic Medicine Institute, Cleveland Clinic, Cleveland, OH, USA; 2 Department of Pathobiology, Cleveland Clinic, Cleveland OH, USA

**Keywords:** pulmonary hypertension, endothelial cells, karyotype, genomic stability

## Abstract

Pulmonary vascular remodeling, including proliferation and migration of pulmonary artery endothelial cells (PAEC), is a pathologic hallmark of pulmonary arterial hypertension (PAH). Multiple studies have shown evidence of increased levels of DNA damage and lineage-specific genetic changes in PAH lung vascular cells, suggesting increased genomic instability. Highly proliferative endothelial colony-forming cell (ECFC) clones can be isolated from PAEC. Here we utilized ECFC to track chromosomal copy number of 20 PAH and eight control clones across serial passages using genome-wide microarrays. All PAH clones were genomically stable for at least 20–22 population doublings. At very late passages, ECFC developed a highly aneuploid karyotype, but this was generally associated with senescence and was common to both PAH and controls. We also utilized ECFC to isolate the chromosomally abnormal cells from a mixed population of PAH PAEC. Analysis of PAEC harboring two different changes affecting chromosomes 1 and X demonstrated that both abnormalities were present in the same clone, indicating they originated in a common ancestral cell. In a second case, with a partial duplication of chromosome 17, clones carrying the duplication were more frequent at later passages than chromosomally normal clones from the same PAEC culture, suggesting the rearrangement may confer a proliferative advantage. Overall, this small study suggests that endothelial cells from PAH lungs are stable in culture, but that when chromosome abnormalities do occur, they may confer a selective advantage that allows expansion of the abnormal cell population and could contribute to lung vascular remodeling in vivo.

## Introduction

Pulmonary arterial hypertension (PAH) is characterized by a progressive increase in pulmonary vascular resistance, which may eventually lead to right ventricular failure.^1,2^ Although an imbalance between pulmonary artery vasoconstriction and vasodilation contributes to the pathophysiology of the disease, it is now widely recognized that remodeling of small pulmonary arteries represents the main pathologic finding related to PAH, with marked proliferation of pulmonary smooth muscle and the formation of plexiform and concentric lesions comprising proliferative endothelial cells and myofibroblasts.^1,3^

Endothelial cells within the plexiform lesions of idiopathic (IPAH) and associated PAH (APAH) cases were previously found to be monoclonal^4,5^ and some lesions also showed microsatellite instability.^6^ There is growing evidence that PAH lung vascular cells show higher levels of DNA damage than controls,^7–10^ and chromosomal abnormalities are detectable in pulmonary artery endothelial cells (PAEC) from explant lung tissue in about one-third of PAH cases.^10,11^ Together, these data suggest PAH may resemble a neoplasia-like process,^12,13^ with the accumulation of multiple genetic mutations. However, it has been difficult to study these abnormalities and their functional effects in detail, as they are often present as a minor clone comprising less than 50% of the overall cell population.

Endothelial colony-forming cells (ECFC) are highly proliferative endothelial cells that have been isolated from the circulation, aorta, arteries, and microvessels.^14–16^ ECFC express endothelial cell-specific markers such as CD34, CD146, CD31, Flk-1, and CD105 and demonstrate the capacity to form functional vessels in vivo.^14,15,17^ Duong et al. were recently able to exploit the proliferative potential of ECFC to generate clonal cultures from PAEC.^18^ Here we utilized this ECFC sub-culture method to isolate and expand clonal populations of endothelial cells from mixed populations of PAH PAEC for detailed genetic analysis. Given the evidence for DNA damage and chromosomal abnormalities in PAH PAEC,^7–11^ we hypothesized that their genomes may be inherently less stable than control cells. Analysis of ECFC allowed us to study the genomic stability of PAH and control clones across multiple passages, and provided new insight into a case with two chromosomal changes.

## Materials and methods

### Study population

PAECs were obtained from the explant lungs of PAH patients and controls (donor lungs unsuitable for transplant), as previously described^19^ and maintained in complete endothelial growth media-2 (EGM-2; Lonza, Walkersville, MD, USA). Analysis for mutations in the *BMPR2* and *SMAD9* genes was performed by direct sequencing and multiplex-ligation dependent probe amplification.^20,21^ For ECFC isolation, we selected PAECs from three patients with different chromosome alterations and one normal control (Table 1). For subsequent DNA analysis, we also included samples from clones derived in a previous experiment.^18^ Numbering of the cases from the previous paper has been preserved to facilitate cross-referencing.

**Table 1.** Characteristics of PAEC used to isolate ECFC.

Case	Clinical diagnosis	*BMPR2* mutation	Somatic chromosome abnormality	Clones analyzed
PAH-3	IPAH	None	None	2
PAH-9	IPAH	None	del(X)	2
PAH-7	PVOD	966A > T	dup(17)(q22-qter)	13
PAH-11	APAH	None	del(1)(q23.2;q23.3); del(X)	1
PAH-12	IPAH	exon 1-8 deletion	del(13)	2
Control	Control	None	None	8

APAH, associated pulmonary arterial hypertension; IPAH, idiopathic pulmonary arterial hypertension; PVOD, pulmonary veno-occlusive disease.

### Endothelial colony-forming cell assay

ECFC were isolated as previously published.^18^ Briefly, primary PAEC were sorted by FACS using a FACSAria II flow cytometry cell sorter (BD Biosciences, San Jose, CA, USA) to place a single cell in each well of a collagen-coated 96-well tissue culture plate. Position A1 of each plate received 100 cells as a positive control and position H12 was the negative control. Six plates were prepared from each case, a total of 564 individual cells. These cells, designated clone passage 0 (CP0), were maintained in EGM-2 supplemented with 10% fetal bovine serum (FBS) and examined for the formation of ECFC colonies by light microscopy after 14 days. For the purposes of our experiments, only colonies that covered at least one-eighth of the well were scored. Colonies covering at least one-third of the surface of a 96-well plate were expanded for further analysis, as described below. Colonies that were not grown further were harvested for DNA analysis by proteinase K digestion at 37℃ for 30 min, followed by heat inactivation for 20 min at 95℃, and 2 µL of the crude lysate was used for polymerase chain reaction (PCR).

### Expansion of ECFC clones

Initial expansion of ECFC colonies was performed as described.^18^ All tissue-culture plates were coated with 5 µg/cm^2^ rat tail collagen-I and cells were maintained in EGM-2 with 10% FBS. Colonies from 96-well plates were trypsinized and transferred to a 24-well plate and designated CP1. Wells in which cells expanded to at least 80% confluence were subsequently sub-cultured to a six-well plate, designated CP2. Confluent wells were then sub-cultured in a 10-cm tissue culture plate, CP3. Previously, PAEC-derived ECFC have not been cultured beyond this point,^18^ but we continued to expand confluent 10-cm plates to later passages by splitting 1:5 until they ceased to divide. All clones reaching the CP4 stage (five 10-cm plates) had one plate harvested for DNA extraction (DNA Mini kit, Qiagen, Valencia, CA, USA), together with a second DNA sample harvested when the cells ceased to divide. Colonies that failed to reach CP4 were extracted at their latest passage using the Qiagen mini or micro kit, or crude proteinase-K digestion method described above, according to the number of cells.

### Analysis of genome-wide copy number changes

To detect genome-wide copy number changes, DNA was hybridized to single nucleotide polymorphism (SNP) arrays (CytoSNP-12 v2, Illumina, San Diego, CA, USA) according to recommended protocols and analyzed using the manufacturers’ software. Unsorted primary cultures were used as a baseline for comparison of ECFC array profiles. Copy number abnormalities were identified from intensity values across the array using GenomeStudio software (Illumina) with the CNV-Partition algorithm and confirmed by observation of the B-allele frequency, which should cluster around 0.5 for heterozygous SNPs with normal copy numbers, but deviates when one allele is deleted or duplicated.

### Chromosome 17 microsatellite analysis

The status of 17q duplication in clones from PAH-7 was determined using microsatellite markers on the long arm within the duplicated region (D17S1290; 17q22) and as a control, on the short arm of chromosome 17 (D17S2196; 17p11.2). PCR was performed using one primer labeled with 6-FAM fluorescent dye and one unlabeled. Samples were heat denatured in formamide and run on a 3730xl genetic analyzer (Applied Biosystems, Foster City, CA, USA) using an in-lane size standard for accurate sizing. Data were analyzed using the Genemapper software (Applied Biosystems). Both markers were heterozygous in PAH-7 and the relative peak height ratio of the two alleles was determined for each clone.

### Metaphase spreads

For preparation of chromosome spreads, ECFC grown to 85% confluence were treated with 0.1 µg/mL colcemid (Karyomax, Invitrogen, Carlsbad, CA, USA) for 2 h to block cells in metaphase. Mitotic cells were removed with 0.1% trypsin, centrifuged, and the medium was aspirated. Pellets were vortexed and 5 mL of hypotonic solution (75 mM KCl; 4℃) was added while vortexing. Samples were then held at room temperature for 10 min before centrifuging again. Hypotonic solution was aspirated, pellets vortexed again, and 5 mL of fixative (methanol:acetic acid, 3:1, made up freshly) was added during vortexing. Tubes were allowed to stand for 10 min before centrifuging again, fixative aspirated, and then 5 mL of fresh fixative added during vortexing. This procedure was repeated four times, after which the cells were transferred to 1.5 mL tubes in 0.5 mL of fixative. Ten microliters of cell suspension were then dropped on to ice-cold ethanol-washed slides and air-dried. Once dry, slides were mounted with 1 µg/mL 4′,6-diamidino-2-phenylindole in Vectashield and visualized by microscopy. Slides were scanned for metaphase cells and the number of chromosomes in 50 metaphase cells was counted.

## Results

### Proliferative potential of ECFC clones

**Fig. 1.**
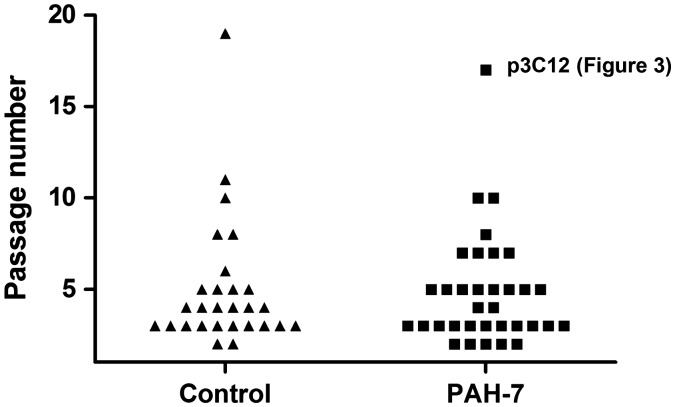
Expansion potential of ECFC colonies. Each symbol represents an individual ECFC clone derived from control (triangle) or PAH-7 (square) PAEC. Vertical axis represents the passage at which each individual clone stopped dividing; initial seeding of individual cells was designated clone passage 0 (CP0). For clarity, only clones that were expanded beyond passage CP2 are included. No significant difference was observed between the expansion potential of control or PAH ECFC.

Cloning of passage 5 PAEC from one control and one patient (PAH-7) yielded ECFC colonies in 108/564 (19%) of wells from the control and 119/564 (21%) from PAH-7. In each case, the 48 largest colonies were transferred to 24-well plates for further expansion. Of these, 28 control and 34 PAH-7 colonies reached 80% confluence and were grown further. Fig. 1 shows the expansion potential of these colonies, indicating the passage at which clones stopped growing. Overall, more than half of the clones reached at least CP3 (approximately 1 million cells), 30% reached CP4 (5 million cells) and a few clones can expand for many more generations, providing sufficient cells for functional analysis.

### Genomic stability of ECFC

To determine whether clones from PAH PAEC accumulate chromosome abnormalities at a faster rate than control cells, we analyzed all of the ECFC colonies that reached CP7 or beyond and compared this with DNA extracted from the same clones at CP4. We later also extracted DNA from cells frozen back at intervening passages in order to study the evolution of these clones over time. In addition, we randomly selected a subset of other clones from these two cases that had stopped growing at earlier passages, and included DNA from seven clones previously isolated from four other PAH cases (Table 1).^18^ Four of the five PAH cases were chosen because they had a pre-existing chromosome abnormality in the parent PAEC culture, while the fifth case and the control were karyotypically normal (Table 1). In total, molecular karyotyping was performed using SNP array analysis on 28 clones, ten of them over multiple passages, to identify any novel chromosome changes not present in the parent culture (Supplementary Table 1).

**Fig. 2.**
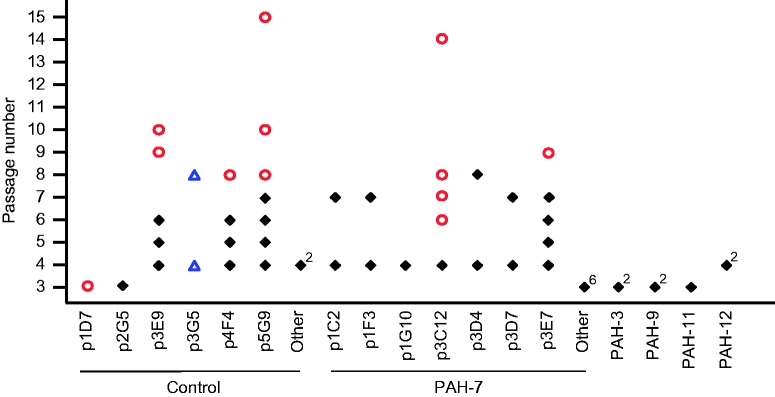
Genomic stability and evolution of ECFC clones. SNP array genotyping was performed on 28 clones from five patients and the control; ten clones were studied over multiple passages. Only abnormalities that were acquired during the growth of the clone were scored; changes known to pre-exist in the parent PAEC culture were not counted as abnormal. Normal genotypes at each data point are indicated by black diamonds, aneuploidy is indicated by red circles. Control clone p3G5 exhibited loss of heterozygosity on 1q, but the overall copy number was normal. This balanced rearrangement is indicated by blue triangles. Superscript numbers indicate multiple independent clones that showed a normal genotype at that passage.

**Fig. 3.**
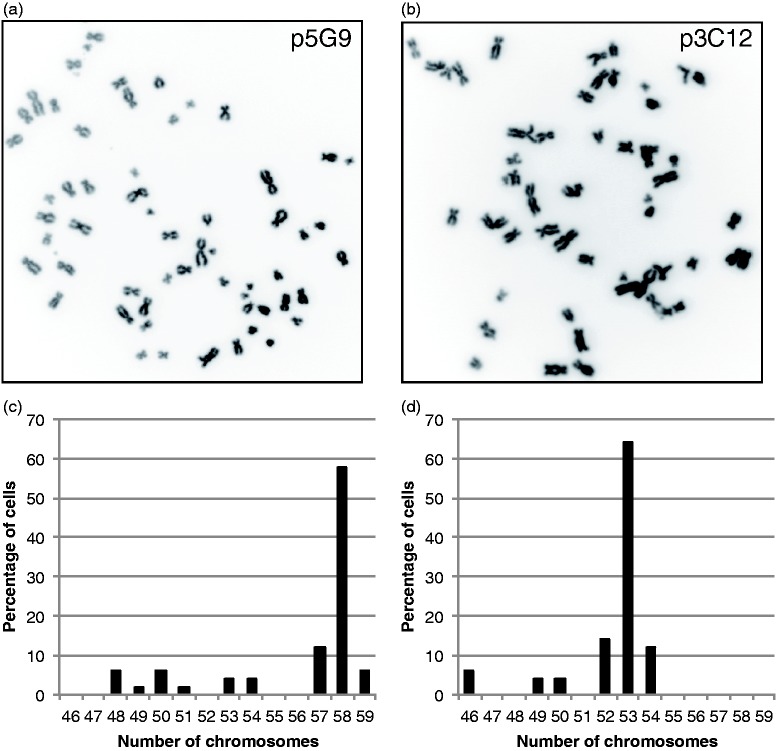
Metaphase spreads of clones with acquired chromosomal abnormalities. (a) Inverted DAPI image of a metaphase spread from control clone p5G9 at passage 17 showing 58 chromosomes and (b) from PAH-7 clone p3C12 at passage 16 cells showing 53 chromosomes. Fifty metaphase spreads were scored for each clone and distribution of the number of chromosomes per cell was plotted (c, d).

At CP3/CP4, new chromosomal abnormalities were only identified in two of the 28 clones, both of which were from the controls (Fig. 2, Supplementary Table 1). Only one abnormality led to genomic imbalance (clone p1D7), whereas the other was a loss and reduplication event that maintained overall gene dosage and was stably propagated to CP8 without further change (clone p3G5). All other clones were either normal or only carried the abnormality known to be present in the parent PAEC population. Of the ten clones followed beyond CP4, four were still stable at the point where they stopped growing. The remaining six developed a high degree of aneuploidy (Figs. 2 and 3, Supplementary Table 1). In four of these six cases, aneuploidy was associated with senescence, whereas the remaining two clones (control p5G9 and PAH-7 p3C12) gained a highly proliferative phenotype and continued to divide beyond CP15. The PAH-7 p3C12 clone was observed to go through a visible bottleneck at CP6, in which the majority of cells were lost. This coincided with the emergence of the aneuploid clone, suggesting the chromosomal changes conferred a strong growth advantage. Consistent with this, the clone then remained very stable and the chromosome complement was almost identical at CP14 (Supplementary Table 1). Control clone p5G9 showed a similar evolution, though no visible bottleneck occurred. A small proportion of abnormal cells could be detected on the array at CP8, but by CP10, the majority of cells harbored multiple chromosome changes, suggesting a very rapid proliferation of an abnormal sub-clone, which then remained stable through CP15. As shown in Fig. 3, metaphase spreads from p5G9 at passage 17 had a mode of 58 chromosomes and PAH-7 p3C12 at passage 16 had 53 chromosomes, both of which are wholly consistent with the array data.

### ECFC expansion allows clonal isolation of genetic sub-populations

**Fig. 4.**
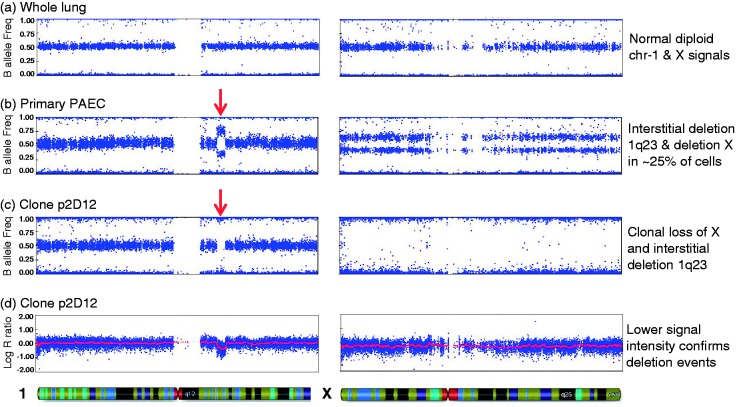
Clonal analysis of PAEC harboring multiple somatic changes. Single nucleotide polymorphism (SNP) array analysis of whole lung tissue from case PAH-11 demonstrated a normal profile, with the B-allele frequency for heterozygous SNPs clustered around 0.5 (a). In primary PAEC, the B-allele ratio deviates from 0.5, indicating an interstitial deletion at 1q23.3-q23.3 (arrowed) and loss of one copy of the X chromosome in a subset of cells (b). ECFC clone p2D12 carries both deletions in 100% of cells, as evidenced by a complete loss of heterozygous SNPs (c) and reduction in log-R ratio, a measure of signal intensity on the array (d).

Clonal expansion of ECFC colonies also enables the isolation of genetically abnormal sub-clones from a PAEC population, allowing a more detailed analysis of the genetic changes. One case of particular interest was a patient with PAH associated with systemic lupus erythematosis, in which we detected two independent genetic alterations in PAEC, a small interstitial deletion at chromosome 1q23.2-q23.3 and complete loss of one copy of the X chromosome (PAH-11, Fig. 4b). Whole lung tissue from the same individual showed no detectable abnormalities, indicating that these are acquired somatic events (Fig. 4a). The estimated proportion of abnormal cells in the PAEC culture was 20–30% for each of the two abnormalities, but it was unclear if both changes had occurred in the same cell, or whether they were present as two distinct sub-clones. Only one ECFC clone from two experiments yielded sufficient DNA for SNP array analysis, but this confirmed that both abnormalities were present as clonal changes (Fig. 4c and d). Although we cannot exclude the possibility that these abnormalities occurred sequentially at different timepoints, this confirms that a single cell acquired two different genetic changes, perhaps simultaneously, and then expanded to form a significant sub-clone within the PAEC population.

The observation that some PAEC cultures contain genetically abnormal sub-clones that constitute 50% or more of all cells suggests that these abnormalities may confer a proliferative advantage. Thus, we hypothesized that some abnormalities might confer a selective advantage for clonogenic potential and/or subsequent proliferation of ECFC clones. We tested this by genotyping all available clones from case PAH-7, which carries a high proportion of cells with a 17q duplication in the parent PAEC. DNA from 103 clones was successfully genotyped using polymorphic microsatellite markers on chromosome 17. Fifteen of these (14.6%) had a normal 1:1 ratio of peaks at 17q and 88 (85.4%) were duplicated (2:1 ratio), a frequency similar to the parent culture. Normal clones were most frequent among the smallest colonies not transferred from the original 96-well plate (12/61; 19.7%) and CP1 clones (2/9; 22%), whereas only 1/33 clones harvested at CP2-CP4 was normal. This suggests that clones with a normal chromosome complement were less able to proliferate beyond CP1 (Fisher’s exact test, two-tailed *P* = 0.033).

## Discussion

Cellular heterogeneity within the systemic circulation is well established and segmental variation in nitric oxide production, surface antigen expression, and proliferative potential have been observed in pulmonary endothelial cells.^16,22^ In addition, genetic heterogeneity has been described in respect of chromosomal abnormalities and microsatellite instability, both in primary PAEC isolated from PAH lung tissue and in the ex vivo tissue itself.^6,10,11^ Here, we isolated clonal ECFC from these PAEC to dissect these genetic changes. In one case, this approach enabled us to demonstrate that two independent changes originated in the same precursor cell, which had subsequently proliferated to comprise approximately 25% of the total culture. In case PAH-7, we first identified the partial duplication of chromosome 17 in three of four ECFC clones without knowing its existence in the parent culture, highlighting the utility of ECFC to tease out sub-populations of cells from a mixed culture. This prompted us to perform a second experiment, generating over 100 clones for the detailed analyses described above. The higher proportion of duplication-17q cells at later clone passages supports the hypothesis that some chromosome abnormalities may confer a proliferative advantage.

The primary goal of the study was to test whether cells from the lungs of patients with PAH are inherently more prone to developing chromosomal abnormalities than controls. We deliberately chose to focus on PAEC that already carried a chromosome abnormality, with the rationale that these cells might be “primed” for further rearrangements. Clones from four such PAH cases were compared with one karyotypically normal PAH case and one control. At clone passage 3 or 4, representing approximately 20–22 population doublings after seeding, all 20 PAH clones had the same chromosome complement as the original PAEC. The limit of detection for an abnormality on these arrays is about 10% of cells, meaning that at least 90% of cells in each clone were normal. Abnormalities were only seen in clones that expanded beyond passage 6, where two of six PAH clones became abnormal. In contrast, only eight control clones were analyzed beyond passage 3, but the abnormality rate was higher. This suggests that genomic instability in endothelial cells from PAH lungs is not markedly higher than controls, at least in respect of changes that are compatible with cell survival. Similarly, late-growth endothelial cells isolated from peripheral blood, which are likely derived from the bone marrow and can expand up to 10^19^ cells, show predominantly normal karyotypes in both PAH patients and controls.^15,22–25^ Our conclusions are drawn from a small number of cases and only one control, and subtle differences may have been missed. However, this already represents a large amount of time to propagate and analyze even this relatively small number of clones, so additional studies are difficult to justify in the absence of any obvious difference.

These results might appear to be at odds with the evidence for increased levels of DNA double-strand breaks in PAH PAEC and pulmonary artery smooth muscle cells reported by several groups.^7–10^ However, the analysis performed here would only detect relatively large rearrangements involving 1 Mb or more of DNA. Smaller deletions or insertions are below the resolution of the array, and would require whole-genome sequencing to detect them. Our approach would also fail to detect any damage that is repaired, or leads to cell death before a clone is established. In general, however, high levels of genomic instability are associated with overt malignancy and would be surprising outside of the cancer field. Rather the emerging phenotype in PAH lung vascular cells appears to be one where levels of DNA damage are elevated and chromosome abnormalities may contribute to increased cell proliferation, but the genomes are largely stable and not subject to the large accumulation of rearrangements that contribute to many solid tumors. The timing of these changes in the pathogenesis of PAH remains unknown, though one case suggested evidence of an early event that was widespread throughout the lung.^26^ As the cost of whole-genome sequencing continues to decrease, analysis of more subtle mutations is becoming feasible, and it is likely that future advances in single-cell sequencing will allow detailed characterization of these cell populations without the need for cloning.

## Supplementary Material

Supplementary material
