# Supplementary material for: Genomic stability of pulmonary artery endothelial colony-forming cells in culture
Source: Pulm Circ. 2017 May 12;7(2):421–7. doi: 10.1177/2045893217700901 (PMC5467930; doi:10.1177/2045893217700901)
Supplement: Supplementary material [file PUL901.doc]

**Supplementary Table 1 – molecular karyotyping results of ECFC clones**

| **Case** | **Clone ID** | **Clone**  **passage** | **Abnormality present from parental PAEC** | **Abnormality acquired during clone propagation** |
| --- | --- | --- | --- | --- |
| Control | p1D7 | 3 | none | +1,+2,+4,+5,+7,+8,-9,+11, +13,+14,+16,+21,+22 |
| Control | p2F11 | 4 | none | NAD |
| Control | p2G5 | 3 | none | NAD |
| Control | p3E9 | 4 | none | NAD |
|  |  | 5 | none | NAD |
|  |  | 6 | none | NAD |
|  |  | 9 | none | +1,+2,-3,+4,+7,+8,-9,-13,+15,+17,+18,+19,+20,+21,+22,+XY |
|  |  | 10 | none | same as CP9 |
| Control | p3F6 | 4 | none | NAD |
| Control | p3G5 | 4 | none | copy neutral LOH of 1q |
|  |  | 8 | none | same as CP4 |
| Control | p4F4 | 4 | none | NAD |
|  |  | 5 | none | NAD |
|  |  | 6 | none | NAD |
|  |  | 8 | none | -4,+7,+8,+10,+11,+12, +16,+17,+18,+19,+22 |
| Control | p5G9 | 4 | none | NAD |
|  |  | 5 | none | NAD |
|  |  | 6 | none | NAD |
|  |  | 7 | none | NAD |
|  |  | 8 | none | Subtle changes on 1,13 and 19 |
|  |  | 10 | none | +1,+2,+3,-4,+5,-6,+7,+9,+12,-13,+14,+15,+16,+19,+20 |
|  |  | 15 | none | +2,+3,-4,+5,-6,+7,+9,+10,+12,-13,+14,+15,+16,+19,+20,+22 |
|  |  |  |  |  |
| PAH-3 | p3E3 | 3 | none | NAD |
| PAH-3 | p5F6 | 3 | none | NAD |
|  |  |  |  |  |
| PAH-7 | p1B5 | 3 | dup(17)(q22-qter)* | NAD |
| PAH-7 | p2A5 | 3 | none | NAD |
| PAH-7 | p2C11 | 3 | dup(17)(q22-qter)* | NAD |
| PAH-7 | p3H8 | 3 | dup(17)(q22-qter)* | NAD |
| PAH-7 | p1C2 | 4 | dup(17)(q22-qter) | NAD |
| PAH-7 | p1C2 | 7 | dup(17)(q22-qter) | NAD |
| PAH-7 | p1F3 | 4 | dup(17)(q22-qter) | NAD |
|  |  | 7 | dup(17)(q22-qter) | NAD |
| PAH-7 | p1G10 | 4 | dup(17)(q22-qter) | NAD |
| PAH-7 | p2A3 | 3 | dup(17)(q22-qter) | NAD |
| PAH-7 | p3C12 | 4 | dup(17)(q22-qter) | NAD |
|  |  | 6 | dup(17)(q22-qter) | +2,+5,-7,+8,+9,+10,+11,-13,-17(normal copy),+20,+21 |
|  |  | 7 | dup(17)(q22-qter) | same as CP6 |
|  |  | 8 | dup(17)(q22-qter) | same as CP6 |
|  |  | 14 | dup(17)(q22-qter) | same as CP6, with an additional complex rearrangement of chr 9 |
| PAH-7 | p3D4 | 4 | dup(17)(q22-qter) | NAD |
|  |  | 8 | dup(17)(q22-qter) | NAD |
| PAH-7 | p3D7 | 4 | dup(17)(q22-qter) | NAD |
|  |  | 7 | dup(17)(q22-qter) | NAD |
| PAH-7 | p3E7 | 4 | dup(17)(q22-qter) | NAD |
|  |  | 5 | dup(17)(q22-qter) | NAD |
|  |  | 6 | dup(17)(q22-qter) | NAD |
|  |  | 7 | dup(17)(q22-qter) | NAD |
|  |  | 9 | dup(17)(q22-qter) | Subtle changes affecting 1,3,5,6,7,13,15,17,19,20,21,22 |
| PAH-7 | p3G2 | 3 | dup(17)(q22-qter) | NAD |
|  |  |  |  |  |
| PAH-9 | p2A5 | 3 | none | NAD |
| PAH-9 | p3G8 | 3 | none | NAD |
|  |  |  |  |  |
| PAH-11 | p2D12 | 3 | del(1)(q23.2;q23.3); del(X) | NAD |
|  |  |  |  |  |
| PAH-12 | p3A7 | 4 | none | NAD |
| PAH-12 | p5B11 | 4 | none | NAD |

NAD, no abnormality detected; +, gain of chromosome; -, loss of chromosome; del, deletion; dup, duplication; q, long arm of chromosome; ter, terminus of the chromosome.

*The 17q duplication was not previously detected in the parent PAEC culture, but following the identification of this abnormality in three different clones, we confirmed the presence of the same abnormality in a different aliquot of the PAEC (see also Figure 3).
